# The therapeutic effect of hyperbaric oxygen therapy in patients with severe to profound idiopathic sudden sensorineural hearing loss

**DOI:** 10.1038/s41598-024-53978-1

**Published:** 2024-02-09

**Authors:** Yeso Choi, Sung Jun Han, Sung Kyun Kim, Seok Min Hong

**Affiliations:** Department of Otorhinolaryngology–Head and Neck Surgery, Dongtan Sacred Heart Hospital, Hallym University College of Medicine, #7 Keunjaebong-gil, Hwaseong-si, 18450 Korea

**Keywords:** Health care, Medical research

## Abstract

The optimal treatment for sudden sensorineural hearing loss (SSNHL) is unclear. Hyperbaric oxygen therapy (HBOT) has been suggested as a viable option for treatment of SSNHL as it improves vascular dysfunction. In this study, we evaluated the therapeutic effects of HBOT by retrospectively reviewing the records of 2206 patients with SSNHL. 54 who had received HBOT were selected for the HBOT groups, while 59 age-matched controls who had not were selected for the control groups. The HBOT and control groups were divided into subgroups according to intratympanic steroid (ITS) use. Groups A–D had received oral steroids + HBOT, oral steroids only, oral steroids + ITS + HBOT, and oral steroids + ITS, respectively. Of the 113 SSNHL patients, 21 had diabetes mellitus (DM) (2, 0, 9, and 10 patients in Groups A–D, respectively). There was no notable difference in hearing improvement between patients receiving HBOT and those in the control group. However, among diabetic patients, those who underwent HBOT demonstrated a significant improvement in hearing when compared to the control group. The combination of HBOT and steroids could potentially be beneficial for treating severe to profound SSNHL patients with DM.

## Introduction

Sudden sensorineural hearing loss (SSNHL) is characterized by sensorineural hearing loss of ≥ 30 dB for ≥ 3 consecutive frequencies occurring within 72 h, and may be accompanied by tinnitus or vertigo^1^. The incidence of SSNHL is estimated to be 5–27 per 100,000 population per year, but may be higher because patients with spontaneous recovery often fail to present to a hospital^2–5^. Although several etiologies, including viral and bacterial infections, vascular disorders, autoimmune disease, and inner-ear and central-nervous-system disorders, have been proposed, the pathogenesis and optimal treatment of SSNHL are unclear^6^. The American Academy of Otolaryngology guidelines suggest that steroids and hyperbaric oxygen therapy (HBOT) should be used as the initial treatment of idiopathic SSNHL^7^. HBOT is based on gas laws, such as Henry’s Law, which states that the quantity of gas dissolved in a liquid or tissue is proportional to the partial pressure in contact with the liquid or tissue^8^. At 1 ATA sea-level, the blood oxygen concentration is 0.3 mL per dL, and resting tissues extract 6 mL of oxygen per dL of blood to maintain normal cellular metabolism. Administering 100% oxygen at 1 ATA increases the blood oxygen level to 2.0 mL per dL; at 3 ATA, the blood dissolved oxygen level is 6.0 mL/dL, which can support resting tissues without any contribution from hemoglobin^9,10^. Therefore, HBOT uses a chamber to deliver 100% oxygen at > 1 ATA pressure to supply oxygen to ischemic tissues, such as the cochlea in the case of SSNHL. Furthermore, HBOT preserves the microcirculation by reducing venular leukocyte adherence and inhibiting progressive adjacent arteriolar vasoconstriction^11^. In summary, HBOT exerts multiple effects on the immune system, oxygen transport, and hemodynamics by reducing hypoxia and edema, thereby generating a normal host response to infection and ischemia^8^. HBOT has been used to treat multiple conditions, such as carbon monoxide poisoning, delayed complications of radiation therapy, chronic diabetic wounds, and ischemic injuries. It was first used in the 1960s in Germany and France for the treatment of SSNHL^12^. The use of HBOT for the treatment of SSNHL is based on the hypothesis that vascular disorders, particularly in the ischemia-sensitive cochlea supplied by the labyrinthine artery without collateral circulation, are involved in some cases and may be the final common pathway for hearing loss of various etiologies. The prognostic roles of age and sex in SSNHL are unclear^4,5,13–16^, whereas severe initial hearing loss and delayed treatment initiation are associated with a poor prognosis^4,5,14–17^. Several studies have evaluated the effects of systemic diseases on recovery from SSNHL. In particular, diabetes mellitus (DM) is closely related to microangiopathic ischemia, which is a potential cause of SSNHL. The high prevalence of SSNHL in DM implies a possible association between the two diseases^4,18–21^. Based on the therapeutic effects of HBOT in ischemia and hypoxia, it may also be useful for the treatment of SSNHL in patients with DM, although this has not been evaluated in previous studies. In this study, we investigated the therapeutic effects of HBOT in patients with severe to profound SSNHL, particularly among those with DM, as both conditions are poor prognostic factors.

## Materials and methods

### Patients

The study protocol was approved by the Institutional Review Board of Hallym University College of Medicine, Republic of Korea (IRB number: 2021-06-003). This study is a retrospective study, and consent or waived from subjects does not adversely affect their dignity, rights, and welfare, informed consent was waived by Institutional Review Board of Hallym University College of Medicine, Republic of Korea. All methods were performed in accordance with the relevant guidelines and regulations. We retrospectively reviewed the medical records of 2206 patients with SSNHL who were identified based on International Classification of Disease codes (ICD-10-CM Diagnosis Code H91.2) and presented to Hallym University Dongtan Sacred Heart Hospital between 1 January 1 2018 and 4 July 2022. Of the 2,206 patients, those with hearing loss > 80 dB, as determined by the average of pure tone audiometric air conduction thresholds at frequencies of 0.5, 1, 2, and 3 kHz at the time of initial diagnosis, and those who received appropriate high-dose oral steroids within 14 days, were included in this study. We excluded patients with inaccurate codes, previous ear surgery, Ménière’s disease, trauma, genetic SNHL, tumor of the auditory pathway, inner ear malformations, loss to follow-up, incomplete audiometric records, age < 18 years, or time from symptom onset to treatment > 14 days. Of the 2206 patients, 54 who met the inclusion criteria and received HBOT were enrolled in the HBOT groups, and 59 age-matched patients among the 83 who met the inclusion criteria and did not receive HBOT were enrolled in the control group. Prior to treatment, patients underwent history-taking, physical examination, and pure-tone audiometry (PTA) conducted by a certified audiologist. Inner-ear magnetic resonance imaging was performed for patients with vertigo, neurological symptoms, or prolonged auditory evoked potentials. We performed chest x-ray in all patients that received HBO to rule out bullous long disease. And, patients with severe ear pain during hyperbaric oxygen treatment due to eustachian tube dysfunction or those with claustrophobia were excluded. In the HBOT group, 16 out of 17 patients in Group A (94%) and 35 out of 37 patients in Group C (94.6%) underwent MRI. In the control group, 8 out of 10 patients in Group B (80%) and 39 out of 49 patients in Group D (79.6%) underwent MRI.

**Figure 1:**
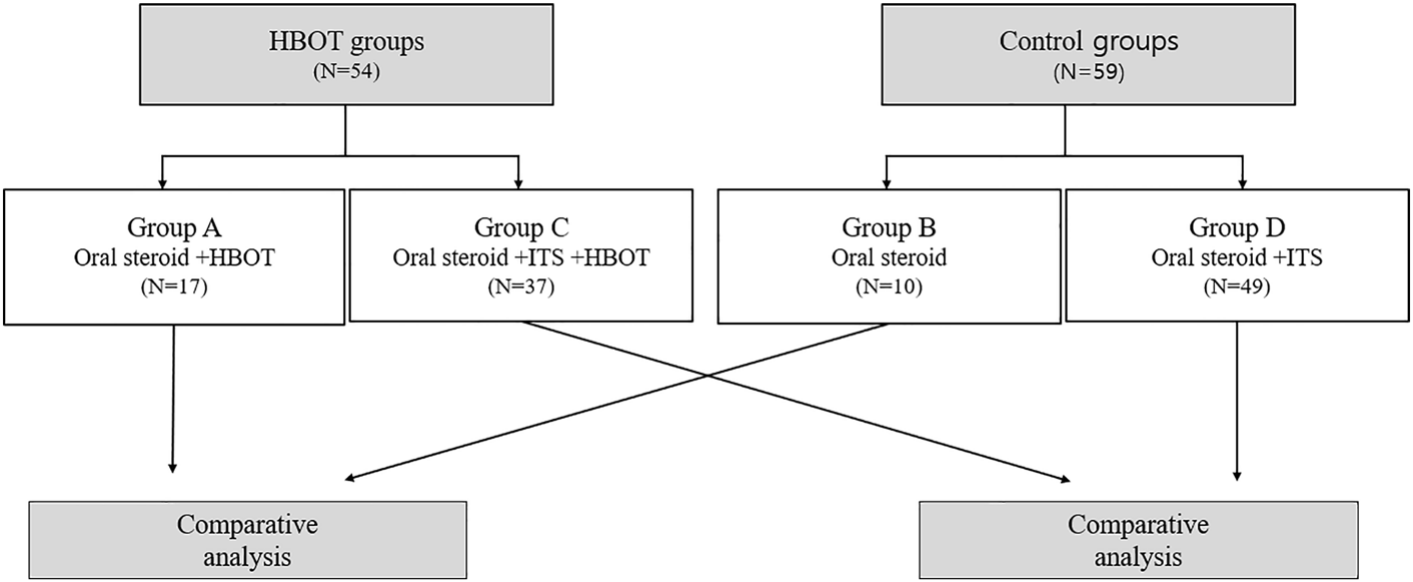
Classification of patients into 4 groups. HBOT, Hyperbaric oxygen therapy; ITS, Intratympanic steroids.

Patients were divided into subgroups based on intratympanic steroid (ITS) use. Groups A–D received oral steroids + HBOT, oral steroids only, oral steroids + ITS + HBOT, and oral steroids + ITS, respectively (Fig. 1). Of the 54 patients who received HBOT, 17 and 37 were included in Groups A and C, respectively. Of the 59 age-matched controls, 10 and 49 were included in Groups B and D, respectively. We compared the age, sex, affected side, time from symptom onset to treatment, initial PTA, and hearing gains between Groups A and B, and C and D. We also compared the effectiveness of HBOT between HBOT and control group patients with diabetes.

### Treatment

All patients received an appropriate dose of oral steroids within 14 days of symptom onset based on their body weight. Patients weighing > 60 kg received 48 mg of methylprednisolone (methylone tablet; Alvogen Korea Co., Ltd., Seoul, Korea) daily for 5 days followed by tapering, whereas patients weighing < 60 kg received 40 mg of methylprednisolone (methylone tablet) daily for 5 days followed by tapering. HBOT was administered daily in a mono-place chamber (IBEX M2; Ibex Medical Systems, Seoul, Korea) or multi-place chamber (IBEX; Ibex Medical Systems) using ventilation with 100% oxygen for 90 min per session at 2.4 ATA pressure. HBOT was administered over 14 sessions; the sessions were terminated if hearing recovered completely. ITS was administered once daily for five consecutive days or, in some cases, performed five times with 2–3 days intervals between sessions, considering individual patient circumstances. For ITS, dexamethasone disodium phosphate (5 mg/mL; Daewon Pharmaceutical Co., Ltd., Seoul, Korea) was injected to fill more than half of the tympanic cavity. During the injection, the patient was placed with the head turned to the contralateral side. After the injection, the patient was instructed to maintain their position and avoid swallowing for 30 min.

### Outcomes

The average PTA thresholds of four frequencies (0.5, 1, 2, and 3 kHz) were calculated. PTA was repeated at 1, 2, 4, and 7–12 weeks after treatment. Hearing gain was determined as the difference between the initial and 7–12-week average PTA thresholds. If the PTA threshold prior to 7 weeks was ≤ 30 dB and no additional PTA was performed, the last PTA was used to calculate improvement without being classified as loss to follow-up. Hearing recovery was classified as complete recovery, partial recovery, slight improvement, or no improvement based on Siegel’s criteria (Table 1)^22^.

**Table 1:** Criteria of hearing recovery (according to Siegel’s criteria).

Type	Hearing recovery
I. Complete recovery	Final hearing better than 25 dB
II. Partial recovery	> 15-dB gain and final hearing 25–45 dB
III. Slight improvement	> 15-dB gain and final hearing poorer than 45 dB
IV. No improvement	< 15-dB gain and final hearing poorer than 75 dB

### Statistical analysis

Data were analyzed using SPSS software (version 27.0; IBM Corp., Armonk, NY, USA). Variables were compared using Student’s t-test, the Mann–Whitney U test, chi-square test, and Fisher’s exact test. *P* < 0.05 was considered to indicate statistical significance.

## Results

There were no significant differences in age, sex, time from symptom onset to treatment, initial PTA threshold (80–89, 90–99, and > 100 dB), or presence of DM between Groups A and B, or between Groups C and D. The right side was more frequently affected in Group C than in Group D (Tables 2 and 3).

**Table 2:** Demographics and audiologic features of patients in Group A and Group B.

Characteristic	Group A (Oral steroid + HBOT) (n = 17)	Group B (Oral steroid) (n = 10)	*p*-value
Mean age (years)	51.0 ± 17.0	56.4 ± 18.5	0.477^a^
Gender (Male: Female)	11:6	5:5	0.687^c^
Side (Right: left)	10:7	7:3	0.692^c^
Time from symptom onset of SSNHL to treatment (days)	2.6 ± 2.1	2.3 ± 2.3	0.428^b^
Initial PTA (dB)	99.9 ± 11.4	101.9 ± 12.3	0.679^a^
80–89 dB SSNHL	3 (17.6%)	2 (20.0%)	1.000^c^
90–99 dB SSNHL	4 (23.5%)	2 (20.0%)	1.000^c^
≥ 100 dB SSNHL	10 (58.8%)	6 (60.0%)	1.000^c^
Rate of diabetes (n [%])	2 (11.7%)	0 (0.0%)	

Data are presented as mean ± standard error of the mean, or subject num.

HBOT, Hyperbaric oxygen therapy; SSNHL, Sudden sensorineural hearing loss; PTA, Pure-tone average.

^a^*p*-value were calculated by student-T test.

^b^*p*-value were calculated by Mann–Whitney test.

^c^*p*-value were calculated by Fisher’s exact test.

**Table 3:** Demographics and audiologic features of patients in Group C and Group D.

Characteristic	Group C (Oral steroid + ITS + HBOT) (n = 37)	Group D (Oral steroid + ITS) (n = 49)	*p*-value
Mean age (years)	45.3 ± 13.1	50.0 ± 14.4	0.121^a^
Gender (Male : Female)	22:15	31:18	0.719^d^
Side (Right : left)	24:13	21:28	0.043^d^*
Time from symptom onset of SSNHL to treatment (days)	2.9 ± 2.6	3.0 ± 2.8	0.976^a^
Initial PTA (dB)	98.7 ± 11.3	100.8 ± 12.4	0.417^a^
80–89 dB SSNHL	11 (29.7%)	13 (26.5%)	0.743^d^
90–99 dB SSNHL	9 (24.3%)	11 (22.4%)	0.838^d^
≥ 100 dB SSNHL	17 (45.9%)	25 (51.0%)	0.641^d^
Rate of diabetes (n [%])	9 (24.3%)	10 (20.4%)	0.665^d^

HBOT, Hyperbaric oxygen therapy; SSNHL, Sudden sensorineural hearing loss; PTA, pure-tone average; ITS, Intratympanic steroids.

Data are presented as mean ± standard error of the mean. * *p* < 0.05.

^a^*p*-value were calculated by student-T test.

^d^*p*-value were calculated by chi-square test.

There were no significant differences in mean hearing gain between Groups A and B (53.3 ± 24.5 and 47.0 ± 20.7 dB, respectively; *p* = 0.503; Table 4), or between Groups C and D (39.1 ± 26.3 and 43.3 ± 25.6 dB, respectively; *p* = 0.455; Table 4).

**Table 4:** Mean hearing gains according to initial PTA in Group A and B & Group C and D.

Initial PTA	Group A (Oral steroid + HBOT) (n = 17)	Group B (Oral steroid) (n = 10)	*p*-value
80–89 dB	47.0 ± 38.3	65.5 ± 9.2	1.000^b^
90–99 dB	70.5 ± 13.5	37.5 ± 41.7	0.533^b^
≥ 100 dB	48.3 ± 22.6	44.0 ± 14.9	0.635^b^
Total	53.3 ± 24.5	47.0 ± 20.7	0.503^b^

Initial PTA	Group C (Oral steroid + ITS + HBOT) (n = 37)	Group D (Oral steroid + ITS) (n = 49)	*p*-value
80–89 dB	36.3 ± 30.6	52.6 ± 27.1	0.106^b^
90–99 dB	56.1 ± 19.2	53.6 ± 24.7	0.802^b^
≥ 100 dB	31.8 ± 23.6	33.9 ± 22.5	0.773^b^
TOTAL	39.1 ± 26.3	43.3 ± 25.6	0.455^a^

Data are presented as mean ± standard error of the mean.

HBOT, Hyperbaric oxygen therapy; PTA, pure-tone average; ITS, Intratympanic steroids.

^a^*p*-value were calculated by student-T test.

^b^*p*-value were calculated by Mann–whitney test.

There were no significant group differences in the mean hearing gains of subgroups based on the initial PTA thresholds (80–89, 90–99, and > 100 dB; Tables 4).

There were no significant differences in hearing recovery based on Siegel’s criteria between Groups A and B. or between Groups C and D (Table 5).

**Table 5:** Number and rate of recovery of patients according to Siegel’s criteria in Group A and B & Group C and D.

Siegel’s criteria type	Group A (Oral steroid + HBOT) (n = 17)	Group B (Oral steroid) (n = 10)	*p*-value
I	4 (23.5%)	2 (20.0%)	1.000^c^
I + II	9 (52.9%)	4 (40.0%)	0.516^c^
I + II + III	14 (82.4%)	8 (80.0%)	0.879^c^
IV	3 (17.6%)	2 (20.0%)	1.000^c^

Siegel’s criteria type	Group C (Oral steroid + ITS + HBOT) (n = 37)	Group D (Oral steroid + ITS) (n = 49)	*p*-value
I	6 (16.2%)	15 (30.6%)	0.124^d^
I + II	14 (37.8%)	18 (36.7%)	1.000^d^
I + II + III	25 (67.6%)	33 (67.3%))	1.000^d^
IV	12 (32.4%)	16 (32.7%)	0.983^d^

Data are presented as number (%).

I. Complete recovery, II. Partial recovery, III. Slight improvement, IV. No improvement.

HBOT, Hyperbaric oxygen therapy; ITS, Intratympanic steroids.

^c^*p*-value were calculated by Fisher’s exact test.

^d^*p*-value were calculated by Chi-square test.

**Figure 2:**
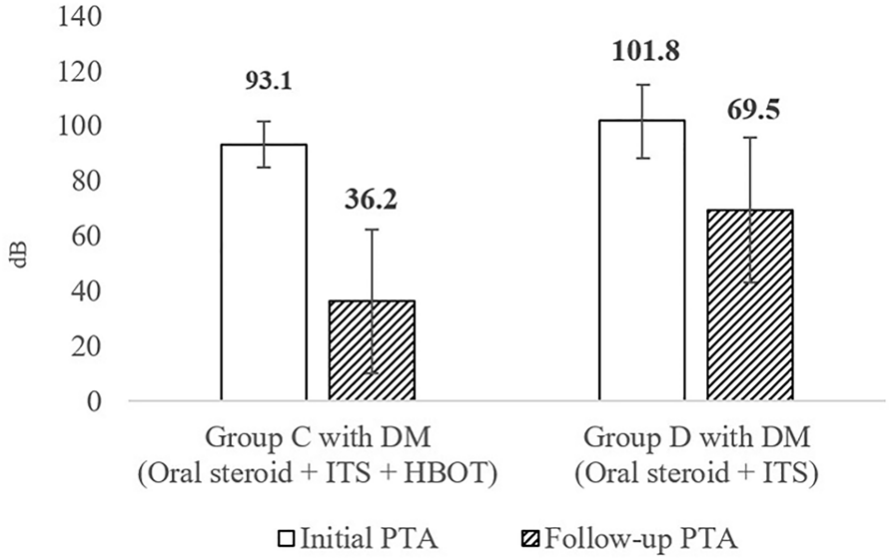
Mean initial PTA and Mean follow-up PTA in Group C with DM and Group D with DM. The mean hearing gains (Follow-up PTA – Initial PTA) of Group C with DM was 56.9 dB ± 20.9 and Group D with DM was 32.3 dB ± 22.5 (*p* = 0.043). DM, Diabetes mellitus; HBOT, Hyperbaric oxygen therapy; ITS, Intratympanic steroids; PTA, Pure-tone average.

Of the 113 SSNHL patients enrolled in this study, 21 (18.6%) had DM. Groups A–D included 2, 0, 9, and 10 patients, respectively. Because none of the patients in Group B had DM, only Groups C and D were compared to evaluate the therapeutic effects of HBOT in patients with SSNHL and DM. There were no significant differences in age, sex, affected side, time from symptom onset to treatment, or initial PTA thresholds (80–89, 90–99, and > 100 dB) between the DM patients in Groups C and D (Table 6). The mean initial PTA thresholds were 93.1 ± 8.3 and 101.8 ± 13.4 dB in Group C and D patients with DM, respectively. The mean follow-up PTA thresholds were 36.2 ± 26.1 and 69.5 ± 26.2 dB in Groups C and D patients with DM, respectively (Table 6, Fig. 2). There were no significant group differences in the initial PTA threshold (*p* = 0.156, Table 6). However, the mean hearing gain was significantly higher in Group C patients with DM than in Group D patients with DM (56.9 and 32.3 dB, respectively; *p* = 0.043; Fig. 2).

**Table 6:** Demographics and audiologic features of patients with DM in Group C with DM and Group D with DM.

Characteristic	Group C with DM (Oral steroid + ITS + HBOT) (n = 9)	Group D with DM (Oral steroid + ITS) (n = 10)	*p*-value
Mean age (years)	47.67 ± 13.629	57.70 ± 10.802	0.092^b^
Gender (Male : Female)	8:1	6:4	0.303^c^
Side (Right : left)	8:1	5:5	0.141^c^
Time from symptom onset of SSNHL to treatment (days)	3.67 ± 2.121	3.50 ± 4.403	0.796^b^
Initial PTA average (dB)	93.11 ± 8.343	101.80 ± 13.390	0.400^b^
80–89 dB SSNHL	3 (33.3%)	3 (30.0%)	0.156^c^
90–99 dB SSNHL	4 (44.4%)	2 (20.0%)	1.000^c^
≥ 100 dB SSNHL	2 (36.8%)	5 (50.0%)	0.350^c^

Data are presented as mean ± standard error of the mean.

DM, Diabetes mellitus; HBOT, Hyperbaric oxygen therapy; SSNHL, Sudden sensorineural hearing loss; ITS, Intratympanic steroids; PTA, Pure-tone average.

^b^*p*-value were calculated by Mann–whitney test.

^c^*p*-value were calculated by Fisher’s exact test.

Complete recovery (Siegel’s criteria type I) was significantly more common in Group C patients with DM than in Group D patients with DM (n = 6 [66.7%] and n = 1 [10%], respectively; *p* = 0.020; Table 7).

**Table 7:** Number and rate of recovery of patients according to Siegel’s criteria in patients with DM in Group C with DM and Group D with DM.

Siegel’s criteria type	Group C with DM (Oral steroid + ITS + HBOT) (n = 9)	Group D with DM (Oral steroid + ITS) (n = 10)	*p*-value
I	6 (66.7%)	1 (10.0%)	0.020^c^*
I + II	6 (66.7%)	2 (20.0%)	0.070^c^
I + II + III	7 (77.8%)	5 (50.0%)	0.350^c^
IV	2 (22.2%)	5 (50.0%)	0.350^c^

I. Complete recovery, II. Partial recovery, III. Slight improvement, IV. No improvement.

Data are presented as mean ± standard error of the mean. * *p* < 0.05.

DM, Diabetes mellitus; HBOT, Hyperbaric oxygen therapy; ITS, Intratympanic steroids.

^c^*p*-value were calculated by Fisher’s exact test.

## Discussion

The spontaneous recovery rate after SSNHL is 65%^3^, whereas the partial recovery rate after severe to profound SSNHL is 20–30%, although the treatment and recovery criteria varied among studies. Byl Jr et al.^4^ reported that 83% of patients with mild hearing loss (< 35 dB) achieved complete recovery, compared to only 22% of those with profound hearing loss. In addition, in a study by Wen et al.^16^ involving 576 patients with profound SSNHL, 172 (29.8%) showed some degree of hearing recovery but only 21 (3.6%) achieved complete recovery. Multiple studies have found that a worse initial PTA threshold correlated with a lower recovery rate^4,5,16,19^. Therefore, we evaluated the therapeutic effects of HBOT in combination with steroids in patients with severe to profound SSNHL.

A Cochrane review^12^, which conducted a meta-analysis on 7 literatures, found no significant benefit of HBOT in SSNHL patients when using a 50% improvement in PTA as the outcome, but found a significant benefit when a 25% improvement was used. The mean improvement in PTA was significantly higher (by 15.6 dB) in the HBOT group than in the control group. However, in our study, the combination of HBOT and steroids was not associated with a higher recovery rate than steroids alone. The disparity in outcomes between our study and the literature may be attributed to the specific population we targeted. Our participants had severe to profound SSNHL, while other studies in the literature included patients with varying degrees of SSNHL. These differences in patient groups may have influenced the hearing outcomes.

In our study, 80 of 113 patients (71%) with severe to profound SSNHL achieved slight to complete recovery, however small, regardless of treatment type. All patients received oral steroids at an appropriate dose within an average of 3 days after symptom onset, and some received additional ITS and/or HBOT, which may explain the higher recovery rate compared to previous studies. Notably, while previous studies typically reported a recovery rate of around 30%, it’s important to highlight that previous studies included patients whose time from symptom onset of SSNHL to treatment exceeded 14 days. While severe to profound SSNHL is generally considered a poor prognostic factor, our findings underscore the significance of prompt and appropriate treatment in potentially achieving hearing improvement.

Animal studies also reported adverse effects of immediate HBOT in cases of acoustic trauma^23,24^. HBOT leads to increased reactive oxygen species, which are known to have deleterious and lethal effects on hair cells in noise-induced hearing loss (NIHL)^24^. But, NIHL is differentiated from SSNHL by pathophysiology. The criteria for initiating HBOT in patients with SSNHL are not yet well-defined. While some argue that early treatment cannot be accurately assessed, given the potential for spontaneous recovery within 2 weeks, it is generally accepted that initiating treatment early can have a positive impact on hearing recovery^25^. The European Committee for Hyperbaric Medicine recommends commencing HBOT within 2 weeks of the onset of SSNHL^26^. However, recent studies have suggested that starting treatment within 7 days may be more beneficial for hearing recovery^27–29^. In our study, 3 patients in Group A and 11 patients in Group C didn’t initiate HBOT within the first 7 days. Consequently, we cannot exclude the possibility that the timing of HBOT initiation in these patients had some effect on the hearing outcomes.

The relationship between DM and SSNHL remains unclear. However, DM patients have a higher risk of developing SSNHL, and SSNHL patients with DM have more severe hearing loss than those without DM^18–21,30,31^. Weng et al.^21^ found that 44.8% of DM patients with SSNHL had profound hearing loss, and most experienced incomplete recovery. In addition, the recovery rate of DM patients with SSNHL was lower compared to that of non-DM patients^32^. Several studies have demonstrated that DM patients with SSNHL have more severe hearing loss and a poorer prognosis than those without DM, although few studies have evaluated the treatment of these patients, particularly using HBOT. A notable study of albino Sprague–Dawley rats found that hyperglycemia induced cochlear damage, and HBOT alleviated the histological changes associated with diabetes-induced cochlear damage^33^.

The mean hearing gains and complete recovery rates of the DM patients in this study with severe to profound SSNHL were significantly higher with combined HBOT and steroid treatment (Group C) than with steroids only (Group D). These findings imply that SSNHL has a vascular etiology and that HBOT may improve severe to profound SSNHL in patients with DM, by enhancing the host response to ischemia through the effect on immune system and oxygen transport^8^.

One limitation of our study is the relatively small sample size of patients with DM, coupled with the execution of multiple statistical tests, which increases the potential for Type 1 errors. To mitigate this limitation and enhance the robustness of our findings, we plan to increase our sample size in future research. Therefore, further studies with large samples are needed to determine the therapeutic effects of HBOT in DM patients with severe to profound SSNHL according to clinical indicators of DM, such as disease duration, mean fasting and postprandial plasma glucose levels, and glycosylated hemoglobin levels.

## Conclusion

There were no differences in hearing improvement between the HBOT and control group patients with severe to profound SSNHL. However, the hearing recovery of the DM patients in the HBOT group was better for that of those in the control group. Our findings imply that HBOT can be considered as one of the treatment methods for patients with severe to profound SSNHL and DM.

### Supplementary Information

Supplementary Tables.

## Data Availability

The analyzed data are available from the corresponding author upon reasonable request.
